# Consumers’ purchase decision in the context of western imported food products: Empirical evidence from Pakistan

**DOI:** 10.1016/j.heliyon.2023.e20358

**Published:** 2023-09-21

**Authors:** Faheem Bukhari, Saima Hussain, Rizwan Raheem Ahmed, Khurram Ali Mubasher, Meer Rujaib Naseem, Muhammad Rizwanullah, Fouzia Nasir, Faiz Ahmed

**Affiliations:** a Iqra University, Defense View Shaheed-e-Millet Road (Ext), Karachi, 75500, Pakistan; b Department of Business Administration, Karachi School of Business and Leadership, Karachi, 74800, Pakistan; c Faculty of Management Sciences, Indus University, Gulshan-17, Karachi, 75300, Pakistan; d Department of Business Administration, KASB Institute of Technology, Karachi, 74400, Pakistan; e Department of Public Administration, University of Karachi, 75270, Pakistan

**Keywords:** Consumer purchase decision, Consumer behavior, Product attributes, Lifestyle, Western imported food, Religiosity, Brand trust, Subjective norms, PLS-SEM modeling

## Abstract

It is essential to identify consumer purchase behavior to establish and implement effective marketing strategies by Western food chains in Pakistan. By identifying motives, firms can offer extra-value products to their current and potential clients. Thus, this study seeks to understand what drives Pakistani consumers to buy imported Western food. This quantitative study uses A standardized structured questionnaire to collect data from 375 Karachi residents. The researchers use a convenient sampling strategy and analyze the data using PLS-SEM modeling through Smart-PLS 4.0. The findings of this research demonstrate that subjective norms, religiosity, product attributes, brand trust, customer satisfaction, and lifestyle significantly and positively influence consumer purchase intention. The findings also show that consumer purchase intention, lifestyle, and subjective norms significantly and positively correlate with purchase behavior. Finally, the study concludes that purchase intention significantly and positively mediates between exogenous and endogenous variables (purchase behavior). This research has significant theoretical and managerial implications. Local and international marketing professionals who wish to investigate the expanding consumer market in Pakistan can find the study's findings extremely useful. In addition, the outcomes of this research enrich the existing body of consumer behavior literature, which is helpful for future researchers.

## 1 Introduction

An organization aims to develop and offer a product or a service with added value. Therefore, it is crucial to understand consumers' preferences and motives [1,2]. Investing in marketing helps organizations achieve their sales revenue and desired market share, but progressive organizations still need help identifying the right consumer preferences. To provide a unique product, comprehending the target consumers' preferences is essential [3]. The Asian subcontinent, including Pakistan, is experiencing a rising food consumption trend that entices Western food producers to explore such consumer markets. Pakistan is witnessing a significant increase in the consumption of imported Western food, despite being an agricultural nation with abundant food production and 97% of the population adhering to Islam [4]. While there is evidence of a gradual increase in the import of Western food products, expenditure on imported items remains substantial. In 2018, Pakistan imported a staggering $1.37 billions of food items, including coffee, vegetables, and canned goods [5]. Additionally, the State Bank of Pakistan reported an increase in food imports totaling $21.3 billion. These data confirm Pakistan's preference for and consumption of imported foods from the West. Consumers spent 49.9 million US dollars on imported food products in 2017 and 75 million in 2018, indicating a 67% increase in preference for imported Western foods [6]. The government will spend nearly $313 in 2018 importing vegetable oil, pulses, tea, coffee, and chocolates [3,5]. The massive growth and preference for Western imported food items reflect that Muslim consumers are not directly adhering to the religious perspective of moderate spending and are willing to spend on imported food items [5,7]. Moreover, the increase in urban population, education level, socioeconomic class shift, supermarket culture rise, acceptance, exposure, and awareness of Western imported food confirm consumers' preference for such products [6,8]. Despite the availability and recognition of local food products, Pakistan remains a significant market for imported food products, with the government and its consumers eagerly embracing them [4,9,10]. Additionally, the Western world, particularly the Australian government and food producers, has demonstrated a strong desire to investigate an Asian subcontinent nation such as Pakistan, where the consumption and preference for imported food have increased and grown over time [1,3,4].

The study aims to identify the key characteristics influencing Pakistani consumers' purchasing decisions when buying imported Western food products. The researchers collect and analyze data related to the consumer behavior of Pakistani consumers. The insights gained from this study can help firms make more informed decisions about product development, pricing, and marketing strategies to meet Pakistani consumers' needs and preferences. The study's primary objective is to evaluate the factors influencing customers' purchasing intention about imported Western food goods. The researchers are interested in determining whether there is a correlation between product attributes, trust in the brand, consumer satisfaction, religious belief, and purchase behavior and intention. They also aim to evaluate the mediation impact of subjective norms and lifestyle between product attributes, brand trust, customer satisfaction, and religiosity factors contributing to the purchase intention leading to the purchase behavior of Muslim consumers. Through survey data, the research empirically proves that product qualities, subjective norms, brand trust, religiosity, and customer happiness determine buying intention, ultimately leading to purchase behavior. The significance of the study in both academic literature and clinical application is supported by the lack of previous studies from populations with a Muslim majority and their desire for foods imported from the West. Determining the specific factors contributing to Pakistani consumers' acceptance and support of Western food brands would add value to the existing body of research on Muslim consumer behavior [3,8].

The primary purpose of this research is to determine whether there is a correlation between the previously stated criteria and consumers' shopping habits. The findings of this study would be beneficial to international food exporters and producers who seek to explore and engage in the Pakistani market since it will help them to execute a marketing and brand strategy following consumer requests [[8], [9], [10]]. It will allow them to capitalize on the opportunities presented by the Pakistani market. The significance and novelty of the study have several folds as this research article provides a novel conceptual framework, and future researchers might replicate this conceptual model in different regions of the globe and sectors of industries [11]. The market practitioners could also benefit from the study and make effective strategies to promote their food products in Islamic countries. This study will benefit developing nations by examining the reasons behind purchasing imported foods from the West. The in-depth analysis would aid local businesses in refining their product and marketing strategies to enhance all local brands [12]. Since most studies in a similar context are conducted in Western countries, obtaining consumer data from a Muslim-majority country and a developing nation like Pakistan can add exciting facts and information to the existing literature on consumer purchasing behavior [[11], [12], [13]]. In addition, considering the level of consumer awareness, the local government's support, and Western food producers' desire to explore this market, there is a strong case for conducting this research on local food products [11].

The paper comprises section two, which presents a literature review, and section three, which outlines the paper's methodology. Section four details the results and findings, while the remaining sections of the paper focus on discussions, conclusions, implications, limitations, and suggestions for future studies.

## 2 Literature review

### 2.1 Theory underpinning – consumer behavior theory (CB-theory)

Consumer behavior theory (CB theory) studies how customers choose which goods or services to purchase, how they use and dispose of them, and how they assess their general level of satisfaction with the purchasing process. CB theory assumes that consumers are active agents seeking information and making decisions based on their needs, goals, and values rather than merely passive recipients of marketing messages. Fishbein and Ajzen [14] introduced the theory of reasoned action (TRA), which served as the foundation for consumer behavior theory. The TRA is a psychological model that accounts for and forecasts conduct regarding attitudes and arbitrary standards. It contends that a person's actions are a product of their intention to carry out such actions, which is, in turn, impacted by their attitude towards those actions and the subjective norms attached. To extend this theory, Ajzen [15] proposed a more complex explanation of consumer behavior known as the theory of planned behavior. This hypothesis considers attitudes about a product, perceived behavioral control (i.e., how much power they feel they have about buying and using the product), and subjective norms to impact customers' intentions to acquire a product (i.e., the extent to which they feel pressure from others to buy or not buy the product). Along with these beliefs, several psychological, social, and personal characteristics like age, income, and lifestyle can affect consumer behavior, including perception, motivation, and learning. Overall, consumer behavior theory research aims to comprehend the intricate and diverse process of how consumers choose what to buy, why, and how they gauge their level of pleasure with those choices. Understanding these elements can help marketers and companies tailor their goods and services to the demands and preferences of their target market more effectively, thereby increasing success and profitability.

### 2.2 Product attributes

Developing a brand's distinctive identity relies on product attributes that help customers choose a specific product. Various factors impact consumer buying behavior, including the taste and ingredients of a product [13,16]. Product packaging is a crucial factor that influences consumer behavior when it comes to food product purchases. Researchers have conducted numerous studies on the influence of product attributes on consumer behavior, highlighting tangible and intangible product elements, including labeling, taste, country of origin, usability, and aesthetics [9,10]. The attributes of imported food brands directly impact consumer buying behavior. Arsil et al. [17] conducted a study on consumer preferences in Malaysia, which affirmed that consumers are attracted to packaging and look for taste, labeling, and country of origin, mainly the halal logo. In contrast, Kumar and Kapoor [18] emphasized the importance of product labeling as a complement to product attributes, enabling consumers to read about the product and persuade them to purchase it. Nguyen and Wismer [19] also stressed the importance of product decisions and their association with consumer buying decisions. Research further indicates a significant and favorable correlation between buyers and their willingness to pay a premium price for sustainable food attributes [8,20]. After considering the perspectives of numerous scholars, we have developed the following hypothesis.

H1 Product attributes are significantly and positively associated with consumer purchase intention.

### 2.3 Brand trust

Consumers play an imperative role in the food segment, and trust is essential, as it dramatically influences their purchase decision. Trust is embedded with prior user experience, brand name, communication, country of origin, and ingredients [21,22]. Ngo et al. [23] believe that trust is based on the product's attributes that satisfy the customers' needs and convince them to stay loyal to the food brand. Barjian et al. [24] suggest that consumer purchase decisions are strongly linked to brand trust. Several authors have established a positive association between prior purchase, food brand name, country of origin, packaging, labeling, ingredients, halal logo, as well as overall product functionality, and the development of consumer trust leading to purchase decisions [8,10,11,13,[25], [26], [27]]. Therefore, we formulate the following hypothesis in light of the preceding discussion.

H2 Brand trust is significantly and positively associated with consumer purchase intention.

### 2.4 Customer satisfaction

Marketers aim to keep their customers satisfied as they understand that satisfied customers are critical assets. Keeping customers happy persuades marketers to offer more value within the product proposition. Li et al. [22] proved a positive association between consumer purchase intentions and customer satisfaction. Factors like efficacy, performance, quality, price, and ingredients that directly impact consumer purchase decisions also enhance customer satisfaction, as put forward by Suchánek and Králová [27]. Consumer spending on food products also directly influences consumer satisfaction [13,28]. Additionally, customer satisfaction relates to the value-added proposition, production standards, brand name, and country of origin, which enhances overall customer satisfaction and usage [[29], [30], [31]]. Based on the preceding discussion, the following hypothesis is formed.

H3 Customer satisfaction is significantly and positively associated with consumer purchase intention.

### 2.5 Religiosity

Researchers have conducted much research over the last two years on the relationship between religious doctrine and customers' purchase choices [4,8]. Religion and the extent of individual bouts of religiosity play a very significant role in shaping someone's choice towards purchasing, especially in the food sector, where most people follow the same religion, Islam, which impacts their buying decisions [28,32]. Since religiosity plays a crucial role in a customer's buying behavior, it is essential to understand and explore its relationship [33,30]. Therefore, the studies shed prominent light on the subject in this aspect. Muslim buyers aim more towards buying in the Islamic way, and their loyalty to their religion is seen when they decide upon food products, as they have to ensure it is halal [3,28,34]. An exploratory and qualitative study in this field claimed that a country whose inhabitants are mainly Muslims had determined religiosity as one of the main drivers of their buying behavior, especially in food [21,29]. Another study focused on Pakistani Muslims and their buying behaviors before Western food. It showed that the importance of religion, family background, and loyalty to Islam is very prominent when they buy something from it [5,31]. In other research, it was also seen that the connection between religiosity and the decision to purchase could be more evident [32,35]. The study's respondents claimed that they tend to strive towards Western imported food goods and make their choices based on their lifestyle, health, likes and dislikes, economic status, salary, country background, awareness of the brand, and exposure to foreign products [13,33,21,35]. The findings of the study have prompted additional research into the relationship between religiosity and consumer spending, leading to the formulation of the following hypothesis.

H4 Religiosity is significantly and positively associated with consumer purchase intention.

### 2.6 Lifestyle

Researchers have conducted a study in China to examine the influence of lifestyles on people's buying and purchasing decisions for domestic and foreign food goods [5,31]. The study found three lifestyles influencing customer behavior: the experiencers, the risk-takers, and the traditionalists. These qualities in a customer can be considered the characteristics of their personalities from a Western perspective. Therefore, it is essential to explore and assess the cross-cultural factors of customer qualities [9,21,30]. The study also found that risk-takers and traditionalists were inclined to purchase imported food [29,32]. Another study has shed light on the model that links food with lifestyle, which includes five behaviors, such as the reasons for buying, the pattern of consuming, the methods of purchasing, the quality factor, and the approaches to cooking [32,35]. Nair's research [35] found that a food-based lifestyle improves the connection between lifestyle, health outcomes, and functional food consumption. According to the study, rational, uninvolved, conservative, adventurous, and careless consumers exist based on their food-based lifestyles [5,13,34]. Interviews showed that about half of the respondents, around 54 out of 90, believed that their lifestyle significantly impacted their purchasing decisions, especially for healthy food. Meanwhile, some respondents, around 12 out of 90, said that their purchasing decisions were based on what they had in mind, regardless of their lifestyle [8,13,36]. In conclusion, the participants emphasized the probable relationship between lifestyles and consumer purchasing decisions, which leads to the formulation of the following hypotheses.

H5 Lifestyle is significantly and positively associated with consumer purchase intention.

H6 Lifestyle is significantly and positively associated with purchase behavior.

### 2.7 Subjective norms

Various aspects impact consumer purchase decisions, and the role of colleagues, friends, and family is equally important. Every individual seeks social approval, influencing their decisions and convincing consumers to decide based on others' perceptions [4,37]. However, prior studies should have incorporated the importance of subjective norms in consumer buying behavior [15,19,37]. On the other hand, some studies have emphasized the notion of subjective norms and their direct influence on consumer buying behavior [20,38]. In their study, Bai et al. [39] confirm that peers, colleagues, and family influence Chinese consumers' buying behavior. Giampietri et al. [40] have proved in one of their studies the influence of the wife, husband, children, and other related family members on consumers' purchase decisions. It signifies that subjective norms and prior approval from peers and family significantly impact consumers' buying behavior [8,13,33]. Based on the previous discussion, the research proposes the following hypotheses.

H7 Subjective norms are significantly and positively associated with consumer purchase intention.

H8 Subjective norms are significantly and positively associated with purchase behavior.

### 2.8 Purchase intention and purchase behavior

Customers' intentions to purchase goods or services are crucial in determining their buying behavior [26,41,42]. Therefore, purchase intention is a significant focus in marketing literature [39,25], as it is directly linked to customer behavior [13,22,30]. Purchase intention plays a crucial role in driving customers to buy a product from the market [2,12,35] repeatedly, and it is an essential factor in customer retention [3,42,43]. Extrinsic factors, such as brand name, price, and convenience, significantly influence customers' purchase intentions [3,42,43]. These findings and literature show that purchase intention strongly influences buying behavior [9,12,28]. As a result, further research is necessary, and the following hypothesis is proposed.

H9 Consumer purchase intention is significantly and positively associated with purchase behavior.

### 2.9 Hypothesized mediating relationship

Several studies have endorsed that purchase intention can mediate the relationship between exogenous and endogenous variables such as Brand trust, Religiosity, Lifestyle, Subjective norms, brand trust, Product attributes, and purchase behavior [13,[44], [45], [46]]. Hence, besides the direct hypothesized relationship of the considered construct, there is also an indirect (mediation) hypothesized relationship. Based on this, the following hypotheses have been formulated.

H10(a) Purchase intention significantly mediates between Brand trust and Purchase behavior.

H810(b) Purchase intention significantly mediates between Customer satisfaction and Purchase behavior.

H810(c) Purchase Intention significantly mediates between Lifestyle and Purchase behavior.

H810(d) Purchase Intention significantly mediates between Product attributes and Purchase behavior.

H10(e) Purchase intention significantly mediates between Religiosity and Purchase behavior.

H10(f) Purchase Intention significantly mediates between Subjective norms and Purchase behavior.

### 2.10 Proposed Conceptual Framework of the study

**Fig. 1:**
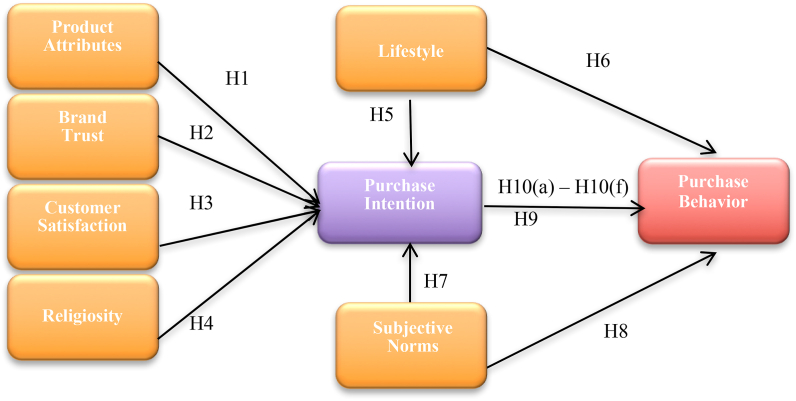
Proposed conceptual framework of the study.

Source: Previous Literature: [2,4,5,8,9,13,15,18,37,[21], [22], [23], [24], [33], [39], [40],[26], [27], [28], [29], [30], [31], [32],^36^,^45^,^46^].

Based on previous literature and discussions, we have proposed and hypothesized the following conceptual framework, shown in Fig. 1 and derived from the previous studies [2,4,5,8,9,13,15,18,37,[21], [22], [23], [24], [33], [39], [40],[26], [27], [28], [29], [30], [31], [32],^36^,^45^,^46^].

## 3 Material and method

### 3.1 Research design

In our quantitative study, we analyze the results using a well-structured survey questionnaire adapted from previous literature to examine the relationship between the proposed variables. We have written the questionnaire in English, Pakistan's second most widely spoken language [4,29]. The questionnaire comprises two interconnected sections. In the first section, we ask participants about their demographic profiles, including age, income level, education, gender, and occupation. The second section of the research instrument uses a five-point Likert scale to ask questions about product attributes, subjective norms, brand trust, customer satisfaction, religiosity, lifestyle, and purchase intentions. We have developed the research instrument from previous research papers, and Appendix I provides the indicators and citations.

### 3.2 Pre-testing or pilot study

The researcher has followed a strict methodological approach, which includes ethical considerations, to ensure the accuracy and reliability of the study. Informed consent has been obtained from every participant before proceeding with the data collection process. The researcher has scanned previous research papers and selected questions that best fit the research objectives to develop the questionnaire [[47], [48], [49]]. After completing the questionnaire, the researcher conducted a pre-test with one industry and subject expert to ensure the correctness of the survey questions. The pre-test results revealed some critical points, and the researcher addressed the issues before proceeding to the pilot testing phase. Fifty respondents, including professionals, homemakers, and university students, participated in the pilot study. The pilot testing results have been appropriate, with a Cronbach value greater than 0.6, ensuring questionnaire reliability [50]. As Yin has suggested, the insights gathered during the pilot study have been used to fine-tune the survey's question sequence.

### 3.3 Data collection method and sampling strategy

In the study, 375 male and female professionals filled out the structured questionnaire, representing an 83% response rate out of 384 respondents. The prior informed consent was obtained from the participants of the study. The study's ethical approval was granted by the Office of Research - Human research ethics committee of the University of Southern Queensland, Toowoomba, Australia (approval number: H16REA237, dated: October 14, 2016, the copy of ethical approval is attached in supplementary material). The structured questionnaire collects data from educated customers older than 18 years old, and the sample size adheres to the parameters established by Hair et al. [51], Ahmed et al. [29], and Hair et al. [52], as the sample size is at least 300. To collect responses, the researchers approached respondents at various periods in the Western food court of shopping malls in Karachi, Pakistan, particularly on weekends. The data collection for this study occurs from March 15, 2022 to July 20, 2022, in Karachi, the largest metropolis in Pakistan. The researchers have gathered the data using a mall-intercept methodology from Dolmen Mall, Lucky Mall, Awami Markaz, Naheed Superstore, and Imtiaz Supermarket. Karachi has a population of 30 million and is densely populated, accounting for approximately 14% of the country's population. The consumption of Western-imported foods is increasing in Karachi, Pakistan, driven by the socioeconomic class, awareness, and consumption of imported food products [4,53]. The researchers have utilized convenience sampling, a nonprobability sampling technique involving the selection of respondents based on specific criteria, such as proximity, accessibility, or inclination to participate [54,55]. This sampling method benefits researchers because it permits the selection of readily accessible participants [55,56]. The study has focused on Karachi, Pakistan, and has collected data for a few months.

### 3.4 Estimation techniques

The researchers have utilized Smart-PLS 4.0 for PLS-SEM modeling in the analysis. PLS examines complex theoretical models that specify relationships between latent variables and associated indicators. In these models, latent variables are concepts that cannot be directly measured but can be inferred from observed indicators related to the underlying construct [52]. PLS-SEM helps analyze these models by enabling researchers to estimate the relationships between latent variables and their indicators while controlling for measurement error and other sources of variance. It achieves this by breaking down the covariance between the observed indicators and the latent variables into a direct and indirect effect mediated by other variables in the model. PLS-SEM has an advantage over traditional SEM because it can handle small sample sizes and non-normal data distributions [55], making it particularly useful in marketing and management research, where sample sizes are often limited, and data distributions often need to be more regular. The researchers have employed various statistical techniques in this study, including Bias Testing and Descriptive Analysis, using Harman's single-factor test as the standard method variance [57]. They have validated the measurement model using Rotated component analysis, factor loadings for each indicator, Cronbach's alpha, Composite reliability, average variance extracted for each construct, Fornell-Lacker criterion, HTMT, and cross-loading. For the validation of the structural model, they used R-square, F-square, Path coefficient analysis, model fitness, and predictive relevance analysis [58].

## 4 Results and data analysis

Through the theory of planned behavior, the study has examined how product qualities, brand trust, lifestyle, customer satisfaction, subjective norm, and religiosity affect buying intention and buying behavior. The quantitative study used 375 respondents and looked for missing values and outliers. The researchers have removed seventy-five cases due to missing data values [57]. Henseler et al. [58] have distinguished the common factor and composite models, focusing on the PLS-SEM method's capabilities as imitators to the CB-SEM method. Both methods evaluate the measurement and structural models using two techniques [51,59,60]. The current research has utilized 375 responses, which is different from the requirement of CB-SEM, which requires a high dataset. The sample size is a vital concern in the CB-SEM technique as it can affect the valuation of the considered model's parameters and its ability to fit the dataset [60]. A larger sample size produces more precise parameter estimates and a better model-data fit [51,61]. Therefore, the study has limitations in using PLS-SEM, the only option due to the shorter sample [55]. However, if a framework has seven or more complex relationships, the 375 responses are enough to analyze the data using PLS-SEM through Smart-PLS 4.0 software. In addition, this research aims to develop a theory through predictive analysis, so the researchers have chosen PLS-SEM to evaluate the framework's explanatory power [52,62]. The researchers have employed several statistical techniques, such as Bias Testing and Descriptive Analysis, to validate the measurement model. They have also used Rotated component analysis, factor loadings for each indicator, Cronbach's alpha, Composite reliability, average variance extracted for each construct, Fornell-Lacker criterion, HTMT, and cross-loading for the same purpose. For the validation of the structural model, they used R-square, F-square, Path coefficient analysis, model fitness, and predictive relevance analysis [58].

### 4.1 Demographic profile of respondents

The findings of Table 1 shows that the total sample comprised 51% male participants. Individuals aged 18 to 24 represented 60% of the sample, and approximately 57% of participants earned a monthly wage between PKR 64,001 to 150,000 (equivalent to US$ 364 to 850). Individuals with a bachelor's degree have the highest levels of education, comprising 53% of the sample, followed by those with a master's degree at 43% and individuals with a Ph.D. at 3%. The sample includes 56% unmarried individuals, and more than 50% of the respondents are full-time employees. Table 2 demonstrates the frequencies and percentages of various behavioral characteristics, including the frequency of procuring imported western food items, the categories of imported western food items procured, the frequency of purchase location, and the primary grocery shopper within the family.

**Table 1:** The demographic profile of the survey participants.

Category	Attribute	Count of Participants	Percentage of Respondents
Gender	Male Candidate	190	51
	Female Candidate	185	49
Age	18–24	225	60
	25–35	89	27
	36–45	50	13
	46–55	07	02
	55+	04	01
Academic qualification	(Undergraduate) Bachelors	200	53
	(Postgraduate) Masters	162	43
	PhD	13	3
Monthly salary in Pakistani rupees	Less than 40,000	10	02
	40,001–64,000	60	16
	64,001–150,000	213	57
	150,001–250,000	85	23
	250,001 +	07	02
Employment status	Permanent	208	55
	Part-time	19	5
	Freelance/Business	37	10
	seeking employment	114	30
Marital Status Marital status	Single	210	56
	Married	165	44

**Table 2:** Respondent's Behavioral attributes.

Item	Categories	Frequency of categories	Percentage
Shopping Frequency	Weekly	52	14%
	Fortnightly	70	19%
	Monthly	198	53%
	Quarterly	30	8%
	Semi-annually	25	7%
Product categories	Chocolates	93	25%
	Baby products	20	5%
	Biscuits	21	6%
	Cereals	46	12%
	Dairy products	38	10%
	Fresh juices	55	15%
	Honey	9	2%
	Ice creams	24	6%
	Imported Fish	10	3%
	Vegetable oil	59	16%
Imported food retail location	Convenience store	45	12%
	General store	41	11%
	Supermarket	272	73%
	Wholesale market	17	5%
Household food shopper	Parents are shopper	90	24%
	Joint Shoppers (Self & Spouse)	212	57%
	Self as a Shopper	73	19%

Table 2 presents the behavioral profile of survey respondents purchasing imported Western foods. The data indicates that 53% of the sample population purchases imported Western cuisines. Chocolates are the most frequently purchased among the imported Western food categories, with 49% of respondents reporting regular purchases. Moreover, fresh juices, cereals, dairy products, and vegetable oil are also popular, with 30%, 25%, 21%, and 18% of respondents reporting regular purchases of these items. Importantly, chocolates were the most frequently imported product from the West, accounting for nearly half of all imports. The survey reveals that supermarkets had a 74% response rate and were the most popular location for purchasing imported Western foods. Furthermore, most respondents (57%) reported that their parents oversaw grocery purchasing for the family. These findings offer valuable insights into consumers' purchasing patterns and preferences regarding imported Western foods.

### 4.2 Statistical analysis

#### 4.2.1 Bias Testing and Descriptive Analysis

The researchers analyzed the data entered and coded in SPSS 26 for outliers using the multivariate outliers' test of Mahalanobis' distance (MD). They found no outliers based on the threshold of 26.13 for eight variables. Descriptive statistics in Table 3 show that the standard deviation, skewness, and kurtosis values of each summated scale are within ±1.5 and ± 3, respectively, confirming the normality of the data. Normality is a prerequisite for using the CB-SEM-based multivariate approach, but in PLS-SEM, normality is not required [55,61].

**Table 3:** Descriptive statistics.

Factors	N	Mean	Std. Deviation	Skewness		Kurtosis	
	Statistic	Statistic	Statistic	Statistic	Std. Error	Statistic	Std. Error
Brand Trust	375	3.78	1.078	−0.895	0.081	0.291	0.164
Customer Satisfaction	375	3.77	1.076	−0.816	0.083	0.196	0.162
Life Style	375	3.83	1.107	−0.956	0.081	0.317	0.161
Product Attribute	375	3.88	1.012	−0.987	0.081	1.022	0.162
Purchase Behavior	375	3.97	1.122	−0.968	0.083	0.277	0.164
Purchase Intention	375	3.83	1.059	−0.936	0.081	0.544	0.162
Religiosity	898	3.94	1.104	−0.952	0.083	0.336	0.161
Subjective Norms	898	3.87	1.084	−0.942	0.081	0.403	0.162

#### 4.2.2 Common method Bias Testing

The researchers have used Harman's single-factor test to examine the standard method variance. They assessed the non-rotated explanation to conclude the number of constructs required to account for the variance of constructs [62,63]. If single construct accounts for most of the covariance among the measures, it concludes that a significant amount of common bias exists [64,65]. The researchers have found that the considered dataset has common method bias because the variance explained increased by 50%. However, Table 4 demonstrates that the considered dataset does not have common method bias because one factor explains only 38.504% of the total variance, which is less than the cut-off value of 50% [63].

**Table 4:** Sampling adequacy and harman single factor.

Comp.	Initial Eigenvalues			Extraction Sums of Squared Loadings		
	Total	% of variance	Cumulative %	Total	% of variance	Cumulative %
1	32.193	38.504	38.504	32.193	38.504	38.504

Extraction method: principal component analysis.

### 4.3 Results of PLS-SEM modeling

After the preliminary dataset evaluation using SPSS 26, the next step is to evaluate and validate the measurement and structural models through PLS-SEM. The first step in PLS-SEM is to validate the considered measurement model [55].

#### 4.3.1 Measurement model

**Fig. 2:**
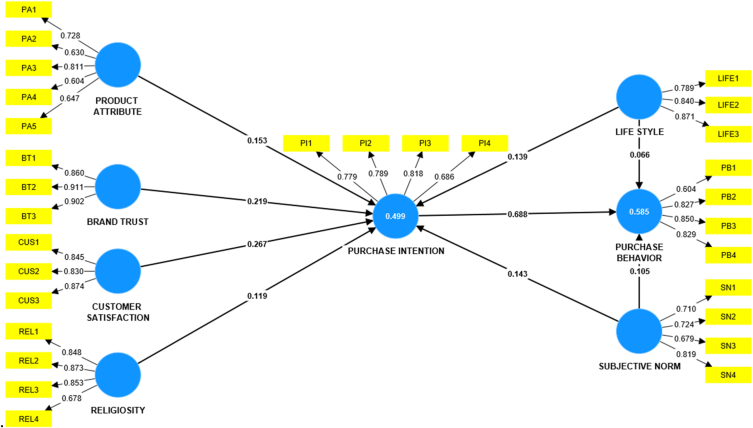
Measurement model of the study.

The researchers have used factor loading of items, Cronbach's alpha, composite reliability, and average variance extracted for constructs to validate the measurement model in PLS-SEM for the validity and reliability of items and constructs. They have established convergent validity between the observed and unobserved variables and discriminant validity between the constructs using the measurement model presented in Fig. 2 [58,60]. The researchers used the Fornell-Larcker criterion and HTMT analysis to evaluate discriminant validity.

***Validity and Reliability of the Data:*** We tested the reliability of the data using Cronbach alpha and composite reliabilities [62], and the values surpassed the threshold values. Table 5 shows that all constructs have composite reliabilities above 0.80 and Cronbach alpha above 0.70. We have verified the model's convergent validity through the average variance extracted, which is above the threshold of 0.50 for all the constructs [66].

**Table 5:** Validity and reliability of the constructs.

Constructs	Cronbach's alpha	Composite reliability (rho_a)	Composite reliability (rho_c)	The average variance extracted (AVE)
Brand Trust	0.870	0.872	0.921	0.795
Customer Satisfaction	0.808	0.814	0.887	0.723
Lifestyle	0.781	0.782	0.873	0.696
Product Attribute	0.718	0.733	0.816	0.501
Purchase Intention	0.769	0.774	0.853	0.592
Purchase Behavior	0.784	0.800	0.863	0.615
Religiosity	0.839	0.888	0.888	0.667
Subjective Norms	0.715	0.723	0.823	0.540

***Outer Loading Matrix:*** During the factor analysis, The researchers removed items with low outer loading <0.60 or high cross-loadings>0.40, particularly from purchase behavior and product attributes [57]. The remaining items in factor analysis have outer loadings greater than 0.60 [67]. Table 6 displays the items selected for construct validity, which confirms the existence of eight variables in the conceptual framework [52,57].

**Table 6:** Outer loading matrix.

	Brand Trust	Customer Satisfaction	Lifestyle	Product Attribute	Purchase Intention	Purchase Behavior	Religiosity	Subjective Norms
BT1	0.860							
BT2	0.911							
BT3	0.902							
CUS1		0.845						
CUS2		0.830						
CUS3		0.874						
LIFE1			0.789					
LIFE2			0.840					
LIFE3			0.871					
PA1				0.728				
PA2				0.630				
PA3				0.811				
PA4				0.604				
PA5				0.647				
PB1						0.604		
PB2						0.827		
PB3						0.850		
PB4						0.829		
PI1					0.779			
PI2					0.789			
PI3					0.818			
PI4					0.686			
REL1							0.848	
REL2							0.873	
REL3							0.853	
REL4							0.678	
SN1								0.710
SN2								0.724
SN3								0.679
SN4								0.819

***Fornell-Larcker criterion:*** The researchers validated the discriminant validity of constructs by assessing the Fornell-Larcker criterion. Table 7 shows that the square roots of each construct's AVEs are more significant than their correlation with any other construct.

**Table 7:** Fornell-larcker discriminant validity.

	Brand Trust	Customer Satisfaction	Life Style	Product Attribute	Purchase Intention	Purchase behavior	Religiosity	Subjective Norm
Brand Trust	0.891							
Customer satisfaction	0.606	0.850						
Life Style	0.205	0.275	0.834					
Product attribute	0.502	0.499	0.237	0.688				
Purchase intention	0.570	0.588	0.320	0.505	0.770			
Purchase Behavior	0.583	0.591	0.300	0.491	0.757	0.784		
Religiosity	0.218	0.111	0.060	0.097	0.250	0.201	0.817	
Subjective Norm	0.405	0.422	0.135	0.449	0.457	0.428	0.212	0.735

***Heterotrait–Monotrait (HTMT) Ratio:*** The findings of Table 8 demonstrate the outcomes of the Heterotrait–Monotrait (HTMT) ratio, which also validates the discriminant validities of constructs. According to Henseler et al. [58], the values of the HTMT matrix are less than 0.85 [51]. Thus, the discriminant validities of constructs are again validated. Hence, the measurement model is validated using PLS-SEM modeling [67].

**Table 8:** Heterotrait–Monotrait (HTMT) ratio.

Constructs	BT	CS	LS	PA	PI	PB	REL	SN
Brand Trust	1.000							
Customer Satisfaction	0.721	1.000						
Life Style	0.249	0.343	1.000					
Product Attribute	0.630	0.652	0.317	1.000				
Purchase Intention	0.693	0.744	0.403	0.674	1.000			
Purchase Behavior	0.699	0.734	0.376	0.642	0.845	1.000		
Religiosity	0.250	0.126	0.099	0.120	0.288	0.235	1.000	
Subjective Norms	0.511	0.544	0.183	0.625	0.611	0.570	0.268	1.000

#### 4.3.2 Structural model

The second stage in PLS-SEM is to validate the structural model; for this purpose, we have used variance inflation factor (VIF), coefficient of determination (R^2^), F-square values, path coefficient analysis, predictive relevance (Q^2^), and overall fitness of the considered model [59,60].

***Variance Inflation Factor (VIF):*** The researchers have opted to test the data biases through inner VIF values. The inner VIF values are less than the benchmark of 3.3. Hence, Table 9 represents the absence of data biases [51,52].

**Table 9:** Inner VIF values.

	Brand Trust	Customer Satisfaction	Life Style	Product Attribute	Purchase Behavior	Purchase Intention
Brand Trust						1.805
Customer Satisfaction						1.819
Lifestyle					1.114	1.099
Product Attribute						1.582
Purchase Behavior						
Purchase Intention					1.383	
Religiosity						1.078
Subjective Norm					1.265	1.399

***Coefficient of determination (R***^***2***^***) and Effect sizes (F***^***2***^***):*** The value of the coefficient of determination adjusted R-square denotes the variation in the purchase intention caused by independent variables and purchase behavior caused by purchase intention. The values are close to 0.50, indicating a moderate level of impact contributed by the independent and intervening variables toward their respective dependent variables [58]. Furthermore, F-square values exceed 0.15. The values of F-square demonstrate the strength of impact on dependent variables caused by the independent [67]. In Table 10, product attributes, brand trust, customer satisfaction, and religiosity strongly influence purchase intention.

**Table 10:** Coefficient of determination (R^2^) and F-Square.

	R Square	R Square Adjusted
Purchase Behavior	0.585	0.584
Purchase Intention	0.499	0.496
**F-Square Effect Sizes**		
	**Purchase Behavior**	**Purchase Intention**
Brand Trust		0.353
Customer Satisfaction		0.178
Life Style	0.459	0.223
Product Attribute		0.454
Purchase Intention	0.826	
Religiosity		1.523
Subjective Norm	0.451	0.221

**Fig. 3:**
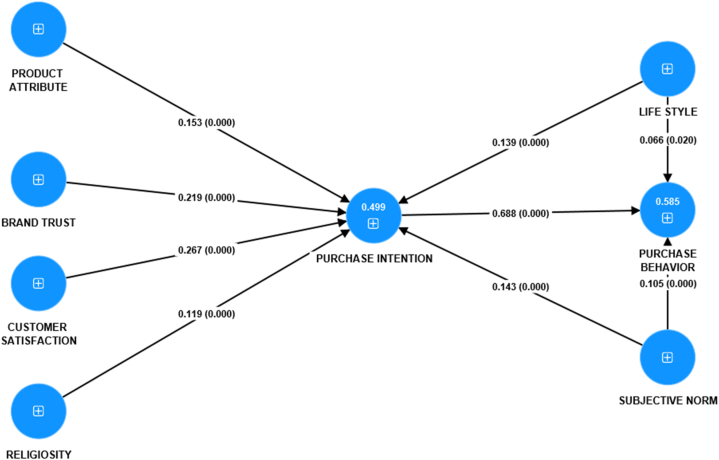
Path analysis of the framework (structural model).

***Framework testing through Path Analysis:*** Smart-PLS 4.0 uses SEM (structural Equation Modeling) for path analysis by bootstrapping 5000 sub-samples [29,52] and testing the final hypothesis in Fig. 3. The path coefficients and acceptance of the hypothesis are depicted in Table 11. The path model in Fig. 3 and analysis in Table 11 verifies the direct relationship between brand trust, customer satisfaction, product attribute, subjective norm, lifestyle, and religiosity toward purchase intention leading to purchase behavior. Lifestyle and subjective norms influence purchase behavior statistically. The individual impact suggests that purchase intention has the most decisive influence on purchase behavior, i.e., β = 0.688, T = 26.74 & P = 0.000. In contrast, customer satisfaction has the second most substantial influence on purchase intention with β = 0.267, T = 6.33 & P = 0.000. Similarly, brand trust significantly and positively influences purchase intention with β = 0.219, T = 6.07 & P = 0.000; however, lifestyle has the lowest influence on purchase behavior with β = 0.066, T = 2.33 & P = 0.020. Hence, H1 to H9 are substantiated because T>±1.96 and P < 0.05.

**Table 11:** Hypothesis testing through path analysis.

Direct Relationship		Original sample (β)	T Statistics	P Values	Result
Brand Trust ->	Purchase Intention	0.219	6.074	0.000	Accepted
Customer Satisfaction ->	Purchase Intention	0.267	6.337	0.000	Accepted
Life Style ->	Purchase Intention	0.139	4.688	0.000	Accepted
Life Style ->	Purchase Behavior	0.066	2.334	0.020	Accepted
Product Attribute ->	Purchase Intention	0.153	4.485	0.000	Accepted
Purchase Intention ->	Purchase Behavior	0.688	26.748	0.000	Accepted
Religiosity ->	Purchase Intention	0.119	4.614	0.000	Accepted
Subjective Norm ->	Purchase Intention	0.143	4.672	0.000	Accepted
Subject Norm ->	Purchase Behavior	0.105	3.972	0.000	Accepted

Table 12 further signifies the indirect impacts of purchase intention on the relationship between product attributes, brand trust, customer satisfaction, religiosity lifestyle, subjective norms, and purchase behavior (dependent variable). Hence, Table 12 shows that purchase intention significantly influences product attributes, brand trust, customer satisfaction, and religiosity purchase behavior. The indirect path verifies the existence of intention leading to the behavior. The findings of Table 12 demonstrate that purchase intention has the highest mediating impact between customer satisfaction and purchase behavior with β = 0.184, T = 6.00 & P = 0.000, and second highest mediating impact between brand trust and purchase behavior with β = 0.151, T = 5.84 & P = 0.000. However, the purchase intention has the lowest mediation between religiosity and purchase behavior with β = 0.082, T = 4.56 & P = 0.000. Hence it is finally concluded that hypotheses H10(a) to H10(f) are substantiated because T>±1.96 and P < 0.05.

**Table 12:** Indirect effects of independent variables on dependent variables.

Indirect Relationship	Original sample (β)	T statistics	P values
CS - > PI- > PB	0.184	6.001	0.000
LS- > PI- > PB	0.095	4.618	0.000
BT- > PI- > PB	0.151	5.849	0.000
SN- > PI- > PB	0.098	4.590	0.000
REL - > PI - > PB	0.082	4.568	0.000
PA- > PI- > PB	0.105	4.439	0.000

Note: BT: Brand Trust; CS: Customer Satisfaction; LS: Lifestyle; PA: Product Attributes; REL: Religiosity; SN: Subjective Norms: PI: Purchase Intention; PB: Purchase Behavior.

*P****redictive Relevance (Q***^***2***^***):*** The findings of Table 13 demonstrate the predictive relevance or Stone-Geisser (Q^2^) of endogenous constructs, for instance, lifestyle, subjective norms purchase behavior & purchase intention, and their respective exogenous constructs, which shows moderately high [58,62]. Thus, it again validates the structural model.

**Table 13:** Predictive relevance (Q^2^).

Factors	Predictive Relevance (Q^2^)
Brand Trust	0.558
Customer Satisfaction	0.431
Lifestyle	0.382
Product Attribute	0.222
Purchase Behavior	0.325
Purchase Intention	0.371
Religiosity	0.452
Subjective Norms	0.240

***Model Fit Indices:*** The researchers have examined the framework hypothesis by conducting principal component analysis in the Smart-PLS 4.0 environment and evaluated the model's quality using SRMR (Standardized Root Mean Square Residual), D_ULS (Euclidean Distance), and NFI (Normal Fit Indices) [58]. Table 14 displays the values of the model fit indices that approach the estimated model's threshold values, indicating that the model is consistent with the hypothesis testing [52].

**Table 14:** Overall model fit indices.

	Saturated Model	Estimated Model	Threshold
SRMR	0.063	0.066	<0.08
d_ULS	1.818	2.050	>0.05
Chi-Square	2140.536	2212.639	
NFI	0.907	0.898	>0.90

## 5 Discussions

The results of the preceding section indicate a significant and affirmative correlation between consumers' purchase intentions for Western food imports and the product's attributes. Thus, it suggests that consumers prioritize product characteristics when making purchases [4,8,68]. This finding is consistent with prior research, such as Alemu et al.'s [16] assertion that product attributes such as packaging, flavor, and freshness substantially affect consumer attitudes toward food products. In addition, Kumar and Kapoor [18] emphasize product safety as a crucial attribute of food brands that substantially impacts consumer attitudes. Furthermore, Nguyen and Wismer [19] discover that established views on product labeling and certification substantially affect consumers' attitudes toward food products. The study also finds that Muslim consumers consider product attributes essential. They associate quality, healthiness, color, labeling, flavor, and shape with the product, which can impact their purchasing decisions. Additionally, the study reveals a direct correlation between the lifestyles of Muslim consumers and their purchase intentions and behaviors for Western imported food products in Pakistan. Therefore, consumers care about flavor, quality, and a health-conscious lifestyle.

Ricci et al. [25] studied consumer perceptions and awareness of imported food products in Italy. They find that consumers prioritize health and pleasure, which contradicts the quantitative findings of the present study. The correlation between subjective norms and consumers' purchasing behavior is more positive and less formal than previously assumed. This finding contradicts the results of Giampietri et al. [40], who discovered a positive association between subjective norms and consumers' food-purchasing behavior. Bai et al. [39] also believe that subjective norms affect food purchases. The present study's quantitative findings indicate a significant positive relationship between subjective norms and customer purchasing behavior, consistent with previous literature. The study also finds that families influence their consumption of high-quality food. In addition, the study examines brand trust and finds a positive and significant correlation between brand trust and consumers' purchase intentions. Previous research by Lerro et al. [21] shows that brand trust is associated with brand image, which reduces the risk for the customer when making a purchase. Therefore, when consumers have a positive impression of a company's brand, they are more likely to have faith in it, increasing customer satisfaction and loyalty. Other studies also report a positive connection between brand image and trust [25,69]. The current study suggests that Muslim customers trust Western imported food products due to their country of origin, brand name, quality ingredients, and production procedures.

Additionally, the Halal label indicating the brand's trustworthiness is significant to consumers. The study also explores the relationship between religiosity and buying intentions and finds a positive and significant correlation consistent with previous research. Previously, Suhartanto et al. [28] pointed out that halal mindfulness and product elements profoundly affect the buying intentions of Muslims. Good practicing Muslims consider Islam their guide to everything in life, and the Quran communicates significant values in deciding their attitudes [3,4,13]. This analysis supports earlier research showing that religiosity dominates the link between relative and contextual characteristics and Muslim consumer purchasing intentions [30,43]. The qualitative interview results suggest Muslims buy based on religiosity, with the halal label and ingredients being the most critical factors. Finally, the study finds that purchase intention significantly mediates between product attributes, brand trust, customer satisfaction, and religious purchase behavior, consistent with previous literature [4,[44], [45], [46]].

## 6 Conclusion

The researchers have illuminated the factors that contribute to the purchase behavior of Muslim consumers in the context of imported food products. They have identified various influencers, including product attributes, subjective norms, brand trust, religiosity, and customer satisfaction. The findings of the study have demonstrated the importance of understanding Muslim consumers' unique purchasing behavior and the significant role of religiosity in their decision-making process. Additionally, the study has provided valuable insights for marketers and food producers, enabling them to devise suitable strategies to target the Muslim community in emerging markets. The research has contributed to the branding literature by establishing the significance of brand trust and product attributes in influencing purchase intention in emerging markets. It has also highlighted the importance of halal certification in the decision-making process of Muslim consumers. The findings indicate that Muslim buyers in Pakistan subconsciously perceive imported food products as high-quality and healthier options in the local market. Muslim buyers assess Western food products through the lens of product attributes, brand trust, customer satisfaction, and subjective norms based on their religiosity traits. The study emphasizes that Islamic marketing and halal food consumption are untapped research areas. The research has identified some major building blocks of purchase intention in the imported food context, such as product attributes, subjective norms, brand trust, religiosity, and customer satisfaction. Furthermore, the study has validated the existence of religiosity, mainly focusing on the dimension of halal ingredients in food products for the followers of Islam. Finally, the study has confirmed that purchase intention mediates between exogenous and endogenous variables. Brand trust exists and affects purchase intention in emergent Muslim markets, adding to branding literature.

### 6.1 Theoretical implications

This study has significant theoretical implications as it contributes to the body of knowledge in several ways. It develops an analytical framework to understand the factors that contribute to the purchase intention of Muslim buyers, empirically verifies the factors affecting the dynamics of consumer behavior, and identifies significant building blocks of purchase intention in the context of imported food. Because there is a dearth of studies on the purchasing decisions made by Muslim consumers in the context of imported food products, this research significantly contributes to the existing body of knowledge. The research creates an analytical framework to understand the elements that contribute to Muslim buyers' purchase intention in emerging countries and empirically verifies the factors that affect the dynamics of consumer behavior. Additionally, the research produces an analytical framework to understand the factors contributing to Muslim buyers' purchase intention in developed markets. This study aims to investigate the factors that influence Muslim consumers' purchasing intentions and behaviors regarding imported food products. The importance of religiosity and halal certification as critical factors that affect consumer behavior is highlighted throughout the study. In addition, the research demonstrates confidence in brands and that this trust substantially impacts consumers' intentions to purchase in rising Muslim markets. This research can help design tactics based on brand trust and religion that local food providers can utilize to persuade Muslim consumers to consume more locally produced food.

### 6.2 Managerial implications

The research findings have significant managerial implications for marketers and food producers who can use them to promote and differentiate their products based on religiosity and halal certification. Local food suppliers can use this research to devise strategies based on brand trust and religion, encouraging local food consumption by Muslims. Western marketers in the Muslim world can also benefit from this study by exploring a diversified market and understanding Muslim consumers' buying behaviors in the sub-continental region. Marketers, brand managers, and communication developers can address the needs of Asian consumers in the context of imported food products by understanding the halal attributes and other quality factors important to Muslim buyers. The research highlights the significance of brand-building activities, as brand trust significantly impacts purchase intention in the case of imported food products. Marketers and importers must study the different dimensions of lifestyle that influence food consumption and purchasing behavior patterns, such as an increase in disposable income, expansion of urban malls, and modernization of grocery retail outlets. Policymakers and strategy formulators of food-importing businesses can utilize the research findings to enhance conscious consumers' awareness by tapping into their religious beliefs and values. The study also brings various opportunities for Pakistani society, particularly local brands competing with imported brands in the food sector. Local food producers can improve their product offerings by adding value in product attributes, ingredients, and packaging to meet the benchmarks of imported food products. They can offer variety through innovative ingredients and healthy options, improving Muslim consumers' overall quality of life.

## 7 Limitations and potential areas of future studies

The study conducted by the researchers has identified certain limitations. The study centers on a solitary element of the marketing mix. It proposes incorporating additional factors into the conceptual framework to examine the purchase intention of Muslim consumers. In order to validate the framework's credibility, it is suggested that it be tested among Muslim consumers hailing from other Eastern regions. Furthermore, demographic factors such as urbanization, gender, age, income, and socioeconomic status may be examined as moderators to augment other aspects of consumer behavior.

Future research may consider classifying religiosity in intra-religious and inter-religious behaviors affecting the purchase decision of imported food products. Although the current research emphasizes the importance of product attributes, religiosity, brand trust, lifestyle, and subjective norms in consumer food purchases, the researchers suggest there is an imperative need to explore the conscious consumption of imported food products in the context of the Pakistani market. They recommend exploring the role of nutritional awareness, food security, and product safety in food consumption in Pakistan. Since the study is conducted in a specific metropolitan city, the researchers suggest that data must be collected from all the major cities of Pakistan to conduct a cross-city comparison and explore the area in depth. They also note that while the study empirically tests various factors leading to consumer purchase intention, it has a few limitations. For instance, the generalizability of the result may be limited due to the data being collected from one metropolitan city. Additionally, consumer behavior is ever-changing, and a cross-sectional survey analysis may need to capture the changes accurately. Therefore, the researchers recommend conducting a longitudinal study with a control group of respondents to analyze the changing behavior of consumers.

## Author contribution statement

Faheem Bukhari: Conceived and designed the experiments; Performed the experiments. Saima Hussain: Analyzed and interpreted the data; Contributed reagents, materials, analysis tools or data; Wrote the paper. Rizwan Raheem Ahmed: Analyzed and interpreted the data; Performed the experiments; Wrote the paper. Khurram Ali Mubasher: Performed the experiments; Analyzed and interpreted the data; Contributed reagents, materials, analysis tools or data. Meer Rujaib Naseem: Contributed reagents, materials, analysis tools or data; Wrote the paper. Muhammad Rizwanullah: Analyzed and interpreted the data; Contributed reagents, materials, analysis tools or data. Fouzia Nasir: Performed the experiments; Wrote the paper. Faiz Ahmed: Contributed reagents, materials, analysis tools or data; Wrote the paper.

## Data availability statement

Data included in article/supplementary material/referenced in article.

## Declaration of competing interest

The authors declare that they have no known competing financial interests or personal relationships that could have appeared to influence the work reported in this paper.

## Figures and Tables

**Table:** 

	Constructs and indicators	Citations
	**PRODUCT ATTRIBUTES**	Bukhari et al. [8]; Lian and Yoong [9]; Kumar and Kapoor [18]; Singh and Verma [37]
1	Western food is healthful.	
2	Fresh imported Western food is valued.	
3	Western food tastes good to many.	
4	Western imported food uses high-quality ingredients.	
5	The halal logo is essential in choosing Western imported food.	
	**LIFESTYLE**	Bukhari et al. [13]; Lerro et al. [21]; Baskaran et al. [31]; Aschemann-Witzel et al. [32]
1	I prioritize eating healthy.	
2	I shop for Western food.	
3	I eat for health and fitness.	
4	I try new Western cuisine products before my friends and neighbors.	
5	I prefer Western-imported cuisine.	
	**SUBJECTIVE NORMS**	Bukhari et al. [4]; Ajzen [15]; Bai et al. [39]; Kaur et al. [33]
1	Friends influenced me to buy imported Western food.	
2	My family affected my Western food purchases.	
3	Colleagues affect my Western food purchases.	
4	Western food keeps family traditions alive.	
5	My family buys imported Western meals.	
	**BRAND TRUST**	Barjian et al. [24]; Ngo et al. [23]; Li et al. [22]; Suchánek and Králová [27]
1	Western food producers are trustworthy.	
2	Western food makers are reliable.	
3	Western-imported food is safe.	
4	Western food sustains me.	
5	Western food importers do not lie.	
	**CUSTOMER SATISFACTION**	Li et al. [22]; Suhartanto et al. [28]; Ahmed et al. [29]; Baskaran et al. [31]
1	I wish to continue buying imported Western meals.	
2	I enjoy eating Western-imported food.	
3	Since I am satisfied, I will pay more for Western imported food.	
4	Western food delights me.	
	**RELIGIOSITY**	Bukhari et al. [4]; Mohammed [5]; Kaur et al. [33]; Patwary et al. [30]
1	Religion addresses many life questions for me.	
2	I live by my religion.	
3	My life is guided by religion.	
4	I like participating in religious events.	
5	I eat halal.	
6	Five times a day, I pray.	
7	I frequently recite the Quran.	
8	I fast every Ramadan.	
	**PURCHASE INTENTION**	Smith and Paladino. [44]; Jin et al. [45]; Liao et al. [46]
1	I plan to suggest Western imported food to my loved ones.	
2	I plan to buy Western food that has been imported.	
3	I plan to opt for Western imported food items as their manufacturers emphasize food safety.	
4	I plan to invest in Western imported food to promote my overall well-being in the long run.	
	**PURCHASE BEHAVIOR**	Gupta and Chitrao [2]; Gkouna et al. [26]; Patwary et al. [30]; Wee et al. [36]
1	I am a frequent buyer of imported Western cuisine.	
2	I prefer to buy Western imported food due to its superior quality.	
3	I prioritize my health by choosing to purchase imported Western food.	
4	I prioritize purchasing Western imported food, regardless of its cost.	
5	I prioritize purchasing Western imported food due to its high safety standards.	
