# Supplementary material for: Consumers’ purchase decision in the context of western imported food products: Empirical evidence from Pakistan
Source: Heliyon. 2023 Sep 21;9(10):e20358. doi: 10.1016/j.heliyon.2023.e20358 (PMC10522991; doi:10.1016/j.heliyon.2023.e20358)
Supplement: Multimedia component 3 [file mmc3.docx]

**Section 1: Demographic profile**

**Instructions**

Please **CIRCLE** on the appropriate choice below:

1. **Please indicate your gender.**

- Male
- Female

1. **What is your age?**

- 18-24
- 25-35
- 36-45
- 46 – 55
- 55+

1. **What is your highest level of education?**

- Primary
- Secondary or (high school)
- Diploma/Vocational education and training
- Undergraduate (Bachelor)
- Post graduate (Masters)

1. **Please estimate your monthly income in Pak rupees.**

- Less than 40,000
- 40,000 – 64,000
- 64,001 – 150,000
- 150,001 – 250,000
- 250,001 +

1. **What is your marital status?**

- Single
- Married
- Other (please specify) _____________

1. **What is your employment status?**

- Full time
- Part time
- Self employed
- Un employed

**Section 2: Behavioural profile**

In this section, please encircle on the answer that best reflects you.

1. **How often do you purchase the western imported food?**

- Daily
- Weekly
- Fortnightly (every 2 weeks)
- Monthly
- Quarterly (every 3 months)
- Semi-annually (every 6 months)
- Other (please specify) __________________

1. **What sort of western imported food do you normally purchase? You may encircle more than one option.**

- Chocolates
- Fresh juices
- Cereals
- Dairy products
- Vegetable oil
- Ice creams
- Biscuits
- Baby products
- Imported fish
- Honey
- Others (please specify) _____________________

1. **Please indicate the type of retail outlet from where you normally buy western imported food? You may encircle more than one option.**

- Super market
- Convenience store (at petrol pumps)
- General store
- Others (please specify) ___________________

1. **Who does the grocery shopping in your family (you can choose more than one option)?**

- Yourself
- Parents
- Jointly (yourself and spouse)
- Other (please specify) __________

**Section 3**

In this section, please answer the following questions by encircle the number which best indicates your opinion about the extent you agree with each statement on a scale from 1 to 5.

**Instruction:** the scale means as follows: 1 = strongly disagree 2 = Disagree 3 = neither agree nor disagree 4 = Agree 5 = strongly agree

|  |  | **Strongly disagree** | **Disagree** | **Neutral** | **Agree** | **Strongly Agree** |
| --- | --- | --- | --- | --- | --- | --- |
|  | **PRODUCT ATTRIBUTES** |  |  |  |  |  |
| 1 | A brand name tells me about the quality of western imported food. | 1 | 2 | 3 | 4 | 5 |
| 2 | Western imported food is healthy. | 1 | 2 | 3 | 4 | 5 |
| 3 | Western imported food is fresh. | 1 | 2 | 3 | 4 | 5 |
| 4 | Western imported food taste good. | 1 | 2 | 3 | 4 | 5 |
| 5 | Western imported food contain better quality ingredients. | 1 | 2 | 3 | 4 | 5 |
| 6 | I trust the claims on the labels of western imported food. | 1 | 2 | 3 | 4 | 5 |
| 7 | The packaging of western imported food gives me a sign about the product quality. | 1 | 2 | 3 | 4 | 5 |
| 8 | The halal logo is important in choosing western imported food. | 1 | 2 | 3 | 4 | 5 |
|  | **LIFESTYLE** |  |  |  |  |  |
| 9 | An important part of my life and activities is to eat healthy food. | 1 | 2 | 3 | 4 | 5 |
| 10 | I usually like to purchase western imported food. | 1 | 2 | 3 | 4 | 5 |
| 11 | I am fitness conscious when it comes to food. | 1 | 2 | 3 | 4 | 5 |
| 12 | I often try western imported food before my friends and neighbours do. | 1 | 2 | 3 | 4 | 5 |
| 13 | To choose between food items, I usually prefer western imported food. | 1 | 2 | 3 | 4 | 5 |
|  | **SUBJECTIVE NORMS** |  |  |  |  |  |
| 14 | My decision to purchase western imported food is influenced by my friends. | 1 | 2 | 3 | 4 | 5 |
| 15 | My decision to purchase western imported food is influenced by my family members. | 1 | 2 | 3 | 4 | 5 |
| 16 | My decision to purchase western imported food is influenced by my colleagues. | 1 | 2 | 3 | 4 | 5 |
| 17 | I buy western imported food to continue family traditions. | 1 | 2 | 3 | 4 | 5 |
| 18 | I buy western imported food because it is an important tradition in my household. | 1 | 2 | 3 | 4 | 5 |
|  | **BRAND TRUST** |  |  |  |  |  |
| 19 | The manufacturers of western imported food are honest. | 1 | 2 | 3 | 4 | 5 |
| 20 | I consider the manufacturers of western imported food to be generally trustworthy. | 1 | 2 | 3 | 4 | 5 |
| 21 | Western imported food is safe to use. | 1 | 2 | 3 | 4 | 5 |
| 22 | I rely on western imported food. | 1 | 2 | 3 | 4 | 5 |
| 23 | I believe the manufacturers of western imported food do not mislead their customers. | 1 | 2 | 3 | 4 | 5 |
|  | **CUSTOMER SATISFACTION** |  |  |  |  |  |
| 24 | I intend to keep purchasing western imported food. | 1 | 2 | 3 | 4 | 5 |
| 25 | I am totally satisfied from western imported food. | 1 | 2 | 3 | 4 | 5 |
| 26 | In the future, I would be willing to pay a higher price for western imported food, since I am a satisfied customer. | 1 | 2 | 3 | 4 | 5 |
| 28 | I consider myself as a satisfied customer of western imported food. | 1 | 2 | 3 | 4 | 5 |
|  | **RELIGIOSITY** |  |  |  |  |  |
| 29 | Religion is especially important to me because it answers many questions about the meaning of life. | 1 | 2 | 3 | 4 | 5 |
| 30 | My religious beliefs lie behind my whole approach to life. | 1 | 2 | 3 | 4 | 5 |
| 31 | Religious beliefs influence all my dealings in life. | 1 | 2 | 3 | 4 | 5 |
| 32 | I enjoy taking part in the activities of my religious group. | 1 | 2 | 3 | 4 | 5 |
| 33 | I only consume halal food. | 1 | 2 | 3 | 4 | 5 |
| 34 | I pray regularly five times a day | 1 | 2 | 3 | 4 | 5 |
| 35 | I regularly recite the holy Quran | 1 | 2 | 3 | 4 | 5 |
| 36 | I fast regularly during Ramadan | 1 | 2 | 3 | 4 | 5 |
|  | **PURCHASE INTENTION** |  |  |  |  |  |
| 37 | I intend to recommend western imported food to family and friends. | 1 | 2 | 3 | 4 | 5 |
| 38 | I definitely intend to purchase western imported food. | 1 | 2 | 3 | 4 | 5 |
| 39 | I intend to purchase western imported food products because their manufacturers are more concerned about food safety. | 1 | 2 | 3 | 4 | 5 |
| 40 | I intend to purchase western imported food for its benefits to my long-term health. | 1 | 2 | 3 | 4 | 5 |
|  | **PURCHASE BEHAVIOUR** |  |  |  |  |  |
| 41 | I purchase western imported food on a regular basis. | 1 | 2 | 3 | 4 | 5 |
| 42 | I purchase western imported food because of its better quality. | 1 | 2 | 3 | 4 | 5 |
| 43 | I purchase western imported food for my health. | 1 | 2 | 3 | 4 | 5 |
| 44 | I purchase western imported food regardless of its price. | 1 | 2 | 3 | 4 | 5 |
| 45 | I purchase western imported food because it is safe to consume. | 1 | 2 | 3 | 4 | 5 |

**Thank you for your time ☺**
