# Supplementary material for: Consumers’ purchase decision in the context of western imported food products: Empirical evidence from Pakistan
Source: Heliyon. 2023 Sep 21;9(10):e20358. doi: 10.1016/j.heliyon.2023.e20358 (PMC10522991; doi:10.1016/j.heliyon.2023.e20358)
Supplement: Multimedia component 4 [file mmc4.pdf]

OFFICE OF RESEARCH
Human Research Ethics Committee
PHONE +61 7 4687 5703| FAX +61 7 4631 5555
EMAIL ethics@usq.edu.au

14 October 2016

Mr Syed Faheem Bukhari
Unit 5
1/3 Uni Plaza Court
Kearneys Spring Qld 4350

Dear Faheem

The USQ Human Research Ethics Committee has recently reviewed your responses to the conditions placed upon the ethical approval for the project outlined below. Your proposal is now deemed to meet the requirements of the *National Statement on Ethical Conduct in Human Research (2007)* and full ethical approval has been granted.

Approval No.	**H16REA237**
Project Title	Exploring Muslim's consumer perception and religiosity towards purchase behaviour in context with western imported food products' in Pakistan
Approval date	17 October 2016
Expiry date	17 October 2019
HREC Decision	**Approved**

The standard conditions of this approval are:

(a) conduct the project strictly in accordance with the proposal submitted and granted ethics approval, including any amendments made to the proposal required by the HREC
(b) advise (email: ethics@usq.edu.au) immediately of any complaints or other issues in relation to the project which may warrant review of the ethical approval of the project
(c) make submission for approval of amendments to the approved project before implementing such changes
(d) provide a 'progress report' for every year of approval
(e) provide a 'final report' when the project is complete
(f) advise in writing if the project has been discontinued, using a 'final report'

For (c) to (f) forms are available on the USQ ethics website:
http://www.usq.edu.au/research/support-development/research-services/research-integrity-ethics/human/forms

Please note that failure to comply with the conditions of approval and the *National Statement (2007)* may result in withdrawal of approval for the project.

You may now commence your project. I wish you all the best for the conduct of the project.

Samantha Davis
Ethics Officer

Copies to: faheemhasanphd@gmail.com
